# Targeted Sequencing in Gastric Cancer: Association with Tumor Molecular Characteristics and FLOT Therapy Effectiveness

**DOI:** 10.3390/cimb46020081

**Published:** 2024-02-01

**Authors:** Liudmila V. Spirina, Alexandra V. Avgustinovich, Olga V. Bakina, Sergey G. Afanas’ev, Maxim Yu. Volkov, Sergey V. Vtorushin, Irina V. Kovaleva, Tatyana S. Klyushina, Igor O. Munkuev

**Affiliations:** 1 Biochemistry and Molecular Biology Division, Siberian State Medical University, 2 Moskovsky Trakt, Tomsk 634050, Russia; irina.kovalyova.kovaleva@mail.ru (I.V.K.); klyushina.tatyana@mail.ru (T.S.K.); mynkyev2002@gmail.com (I.O.M.); 2 Cancer Research Institute, Tomsk National Research Medical Center of the Russian Academy of Sciences, 5 Kooperativny Street, Tomsk 634050, Russia; aov862@yandex.ru (A.V.A.); ovbakina@ispms.ru (O.V.B.); afanasievsg@oncology.tomsk.ru (S.G.A.); dok75-75@mail.ru (M.Y.V.); 3 Institute of Strength Physics and Materials Science, Siberian Branch of the Russian Academy of Sciences, 2/4 Pr. Akademicheskii, Tomsk 634055, Russia

**Keywords:** gastric cancer, targeted sequencing, HER2 status, PD-L1 status, molecular markers

## Abstract

Heterogeneity of gastric cancer (GC) is the main trigger of the disease’s relapse. The aim of this study was to investigate the connections between targeted genes, cancer clinical features, and the effectiveness of FLOT chemotherapy. Twenty-one patients with gastric cancers (GCs) were included in this study. Tumor-targeted sequencing was conducted, and real-time PCR was used to assess the expression of molecular markers in tumors. Seven patients with stabilization had mutations that were related to their response to therapy and were relevant to the tumor phenotype. Two patients had two mutations. The number of patients with TP53 mutations increased in HER2-positive tumor status. PD-L1-positive cancers had mutations in *KRAS*, *TP53*, *PIK3CA*, *PTEN*, and *ERBB*, which resulted in an increase in PD-1 expression. TP53 mutation and PTEN mutation are associated with changes in factors associated with neoangiogenesis. In concusion, patients who did not have aggressive growth markers that were verified by molecular features had the best response to treatment, including complete morphologic regression.

## 1. Introduction

Gastric cancer (GC) is still a significant clinical issue in oncology. Heterogeneity of gastric cancer is the main reason for the patient’s unfavorable outcome [1]. It has been found that the landscape of the GC genome and its intratumor heterogeneity affect the tumor behavior and the patient’s prognosis [2]. According to Globocan, the Russian Federation ranks fifth in the prevalence of GCs in the world, with more than 37,000 new cases of malignant neoplasms detected in the Russian Federation each year in the group of both sexes aged 35 years and older [3].

Some GC molecular features are found to dominate in oncogenesis and determine the following molecular subtypes: Epstein–Barr virus-associated, microsatellite instable tumors, cancer with stable genome, and chromosomal instable cancer [4]. Previously, the most commonly mutated genes in GCs were *TP53* (54%), *ARID1A* (23%), *CDH1* (22%), *PIK3CA* (12%), *RNF43* (10%), and *KRAS* (9%) [5].

However, many research efforts have been concentrated on identifying and understanding the cellular invasive potential of tumors. It is known that the presence of a high mutational load in PD-L1-positive tumors is accompanied by defects in the DNA repair system, which are associated with defects in the following genes: *LRP1B* (79.07%), *ARID1A* (74.42%), *RNF43* (69.77%), *ZFHX3* (65.12%), *TP53* (58.14%), *GANS* (51.16%), *BRCA2* (51.16%), *PIK3CA* (51.16%), *NOTCH1* (51.16%), *SMARCA4* (48.84%), *ATR* (46.51%), *POLE* (41.86%), and *ATM* (39.53%) [6]. At the same time, a spectrum of genes associated with poor disease prognosis has been identified (*FN1*, *COL1A2*, *COL1A1*, *COL3A1*, *COL4A1*, *COL6A3*, *COL5A2*, *SPARC*, *PDGFRB*, *COL12A1*).

Low treatment effectiveness has been obtained in GC patients [7]. Heterogeneity of the molecular mechanisms and revealed resistance to the anti-cancer agents highlight the potential strategies for new targeted therapy that could overcome insensitivity and increase the rate of benefits of the treatment [8].

Previously used histology-based division of GCs according to Laurén classification could not increase both accuracy and effectiveness of the anti-cancer treatment. More efforts have been applied to the inclusion of new molecular classifications of GC in translational clinical studies [9]. Obtaining serum molecules has been demonstrated to hold potential for the development of powerful diagnostic markers, to improve on the low sensitivity demonstrated previously for GC detection [10].

Targeted agents’ usage in anti-cancer therapy could be beneficially applied to block the key molecules in oncogenesis [11]. It has been revealed that the molecular landscape in cancers is determined by signaling pathway components and transactional factors. However, the relationship between genetic alterations and features of tumor behavior has not been studied. It is believed that the cancer transcriptomic factors may reflect features of oncogenesis. GCs with bone metastases were found to have a more diverse genomic landscape than tumors without metastases [12]. In addition, the identified changes were combined with changes in signaling pathway activation. The PIK3/AKT/mTOR pathway might be considered as a potential target of antitumor therapy [13,14]. mTOR mediates PD-L1 expression in GCs [15,16], which was found to be associated with MUC16 mutation [17]. 

The variety of altered genes has a significant impact on GCs. However, this effect is still unclear and needs further exploration [18]. A personalized approach in targeted anti-cancer treatment could be effective due to the involvement of selective molecules with great impacts on oncogenesis [19,20]. Considering the potential of such a treatment approach, the purpose of this study was to investigate the relationship of targeted genetic markers in GC patients with clinical and morphological tumor features, as well as the effectiveness of FLOT chemotherapy.

## 2. Materials and Methods

This study included twenty-one patients undergoing treatment from 2019 to 2023 in the abdominal department of the Cancer Research Institute with GCs (tumor process stage T2-4N0-2M0; the age of patients ranged from 36 to 69 years; the average age was 57.1 years). The exclusion criteria were previous special treatment, cardiac localization of the tumor, distant metastases, primary multiple synchronous and metachronous process (except for basal cell skin cancer), clinically significant comorbidities, individual intolerance to chemotherapy components, and complicated forms of gastric cancer (cachexia, decompensated pyloric stenosis, ongoing gastric bleeding requiring emergency surgery, tumor perforation). Patients received combined treatment according to the FLOT (fluorouracil, leucovorin, oxaliplatin, docetaxel) regimen with the addition of targeted immunopreparations (immunotherapy) (trastuzumab for HER2-positive tumor and pembrolizumab for PD-L1-positive tumor). The effectiveness of treatment was assessed using RECIST 1.1 criteria.

The study materials for molecular marker research were samples of the tumor and unchanged tissue obtained during a biopsy, located at least 1 cm from the tumor border, which were frozen after collection and stored at −80 °C. 

Biopsy samples were collected from all patients at the stage of diagnosis (Table 1). Paraffin blocks of biopsy samples were used for DNA extraction. This work was approved by the Ethical Committee of the Tomsk NIMC Oncology Research Institute. All procedures involving patients were performed in accordance with the Protocol of the Helsinki Declaration of Human Rights (1964; protocol №22 dated 28 November 2022). All patients signed informed consent for participation in the research.

*Targeted sequencing.* Targeted sequencing of human gene regions by paired-end reads was performed using the method of amplification of genomic DNA regions (amplicon panel). A targeting panel consisting of 48 genes was used. Onconetix panel (48 genes): *AKT1*, *ALK*, *APC*, *BRAF*, *CDH1*, *CDKN2A*, *CTNNB1*, *DDR2*, *EGFR*, *EIF1AX*, *ERBB2*, *ERBB4*, *FGFR1*, *FGFR2*, *FGFR3*, *FOXL2*, *GNA11*, *GNAQ*, *GNAS*, *H3F3A*, *HIST1H3B*, *HIST1H3C*, *HNF1A*, *HRAS*, *IDH1*, *IDH2*, *KDR*, *KIT*, *KRAS*, *MAP2K1*, *MET*, *MLH1*, *NRAS*, *PDGFRA*, *PIK3CA*, *PTEN*, *RET*, *ROS1*, *SF3B1*, *SMAD4*, *SMARCB1*, *SMO*, *SRC*, *STK11*, *TERT*, *TP53*, *TSC1*, *VHL*.

DNA from paraffin blocks was extracted using the ReliaPrep™ FFPE gDNA Miniprep System kit (Promega, Madison, WI, USA). COSMIC, ClinVar, and NCBI databases were used for comparative analysis and to search for known mutations. Sequencing was performed on the Novoseq platform.

*RNA extraction.* RNA was extracted using the RNeasy mini kit containing DNAase I (Qiagen, Hilden, Germany). The concentration and purity of extracted RNA were evaluated on a NanoDrop-2000 spectrophotometer (Thermo Scientific, Waltham, MA, USA). RNA concentration ranged from 80 to 250 ng/μL (A260/A280 = 1.95–2.05; A260/A230 = 1.90–2.31). RNA integrity was assessed by capillary electrophoresis on a TapeStation instrument (Agilent Technologies, Santa Clara, CA, USA) and an R6K ScreenTape kit (Agilent Technologies, Santa Clara, CA, USA). RIN was 5.6–7.8.

*Quantitative real-time reverse-transcription PCR (RT-qPCR).* Gene expression levels were assessed by quantitative real-time reverse transcription PCR (RT-qPCR) using SYBR Green on an iCycler (Bio-Rad, Hercules, CA, USA). To obtain cDNA on an RNA matrix, a reverse transcription reaction was performed using the OT m-MuLV-RH kit (BioLabmix, Novosibirsk, Russia) with random hexanucleotide primers according to the kit instructions. PCR was performed in three replicates in a volume of 25 μL, containing 12.5 μL BioMaster HS-qPCR SYBR Blue (BioLabmix, Novosibirsk, Russia), 300 nM forward and reverse primers, and 50 ng of cDNA: *CAIX:* F 5′-GTTGCTGTCTCGCTTGGAA-3′, R 5′-CAGGGTGTCAGAGAGGGTGT-3′; *HIF-1:* F 5′-CAAGAACCTACTGCTAATGCCA-3′, R 5′-TTTGGTGAGGCTGTCCGA-3′; *EPAS1:* F 5′-TGGAGTATGAAGAGCAAGCCT-3′, R 5′-GGGAACCTGCTCTTGCTGT-3′; *NFKB1:* F 5′-CGTGTAAACCAAAGCCCTAAA-3′, R 5′-AACCAAGAAAGGAAGCCAAGT-3′; *RELA:* F 5′-GGAGCACAGATACCACCAAGA-3′, R 5′-GGGTTGTTGTTGGTCTGGAT-3′; *VEGFA:* F 5′-AGGGCAGAATCATCACGAA-3′, R 5′-TCTTGCTCTATCTTTCTTTGGTCT-3′; *KDR:* F 5′-AACACAGCAGGAATCAGTCA-3′, R 5′-GTGGTGTCTGTGTCATCGGA-3′; *4-BP1:* F 5′-CAGCCCTTTCTCCCTCACT-3′, R 5′-TTCCCAAGCACATCAACCT-3′; *AKT1*: F 5′-CGAGGACGCCAAGGAGA-3′, R 5′-GTCATCTTGGTCAGGTGGTGT-3′; *C-RAF:* F 5′-TGGTGTGTCCTGCTCCCT-3′, R 5′-ACTGCCTGCTACCTTACTTCCT-3′; *GSK3b:* F 5′-AGACAAGGACGGCAGCAA-3′, R 5′-TGGAGTAGAAGAAATAACGCAAT-3′; *70S kinase alpha:* F 5′-CAGCACAGCAAATCCTCAGA-3′, R 5′- ACACATCTCCCTCTCCACCTT-3′; *m-TOR:* F 5′- CCAAAGGCAACAAGCGAT-3′, R 5′- TTCACCAAACCGTCTCCAA-3′; *PDK1*: F 5′-TCACCAGGACAGCCAATACA-3′, R 5′- CTCCTCGGTCACTCATCTTCA-3′; *VHL*: F 5’—GGCAGGCGAATCTCTTGA-3´, R 5´-CTATTTCCTTTACTCAGCACCATT-3´; *PD-L2*: F 5′-GTTCCACATACCTCAAGTCCAA-3′, R 5′-ATAGCACTGTTCACTTCCCTCTT-3′; *PD-L1:* F 5′-AGGGAGAATGATGGATGTGAA-3′, R 5′-ATCATTCACAACCACACTCACAT-3′; *PD-1-1:* F 5′-CTGGGCGGTGCTACAACT-3′, R 5′-CTTCTGCCCTTCTCTCTGTCA-3′; LC3B: F 5′-CCCAAACCGCAGACACAT-3′, R 5′-ATCCCACCAGCCAGCAC-3′; AMPK: F 5′-AAGATGTCCATTGGATGCACT-3′, R 5′-TGAGGTGTTGAGGAACCAGAT-3′; *GAPDH:* F 5′-GGAAGTCAGGTGGAGCGA-3′, R 5′-GCAACAATATCCACTTTACCAGA-3′.

The two-step amplification program comprised 1 cycle—10 min at 94 °C for pre-denaturation; 40 cycles—step 1 of 10 s at 94 °C and step 2 of 20 s at 60 °C. Primers were selected using the Vector NTI Advance 11.5 program and the NCBI database (http://www.ncbi.nlm.nih.gov/nuccore; 13 December 2023).

The “housekeeping” gene of the enzyme GAPDH (glyceraldehyde-3-phosphate dehydrogenase) was used as a reference gene, and the expression level of each target gene was normalized with respect to GAPDH expression. Quantitative analysis of gene expression was conducted using the formula 2^−ΔΔCt^ relative to the constitutively expressed GAPDH enzyme reference gene.

*Statistical analysis* of results was performed using the Statistica 12.0 software package. Normality was checked using the Kolmogorov–Smirnov criterion. The results of gene expression determination were presented as Me (Q1; Q3). Significance of differences between independent parameters was evaluated by the Mann–Whitney test. Differences in frequency distributions of qualitative features were evaluated using the χ^2^ criterion.

## 3. Results

### 3.1. The Efficacy/Effectiveness in GC Patients and Association with Genetic Markers 

In this research, regression of the tumor process after the combined treatment was observed in fourteen patients (66.6%), of which five patients had complete regression (23.8%). Stabilization was observed in seven patients (33.3%) (Table 2). Targeted sequencing of forty-eight genes identified six patients (28.6%) with no significant markers associated with response to therapy and no genetic variants relevant to tumor phenotype. It is worth noting that there were no significant differences in the distribution of tumors with the presence of mutations among patients with regression and stabilization of the disease.

Of the fourteen partial regression patients, seven were identified with the presence of mutations associated with response to therapy and relevant to tumor phenotype (two mutations were noted in two patients). It was identified that a *PIK3CA* mutation (chr3:g.178952085A>G chr3:g.178936091G>A) was associated with response to therapy in 22.2% of cases, *TP53* mutation—44.4%, *PTEN* mutation—11.1%, *ERBB* mutation—11.1%, and *MAPK* mutation—11.1% (Table 2).

When the tumor process stabilized, eleven mutations were noted in patients, of which double mutations were detected in four people. A *KRAS* mutation (chr12:g.25398284C>T) was recorded in 9.0% of cases; a *PIK3CA* mutation (chr3:g.178952085A>G chr3:g.178936091G>A) was associated with response to therapy; 27.3% had a *TP53* mutation; 9.0% had a PIK3CA mutation; 9.0% had a PTEN mutation; 9.0% had a SMAD4 mutation; and 9.0% had a CTNNB mutation.

Heterogeneity of gastric cancer is the main reason for the development of an unfavorable disease outcome [1]. Currently, knowledge is building about the connection of the genomic landscape of the tumor to its clinical features and treatment efficacy [2].

It is likely that the response to antitumor treatment may be related to the genomic landscape of the tumor [5]. In particular, a better response to treatment has been associated with the absence of significant mutations, as demonstrated in those with a complete response.

### 3.2. Genetic Markers in GC Patients Depending on HER2 and PD-L1 Status 

HER2-positive status was detected in four patients, of whom two had no markers associated with response to therapy and relevant to tumor phenotype, and two had identified mutations in the *TP53* gene (Table 3). PD-L1-positive status was detected in five patients, with a *KRAS* mutation in 20% (of cases), *TP53* mutation in 20%, *PIK3CA* mutation in 20%, *PTEN* mutation in 20%, and *ERBB* mutation in 20%.

It is known that the presence of a high tumor mutation burden (TMB-H) in PD-L1-positive tumors is accompanied by defects in the DNA repair system [6,21]. This fact is consistent with our data, which showed a PD-L1-positive tumor status was characterized by heterogeneity and diversity of the mutation spectrum in the tumor. It should be noted that in patients with different HER2 and PD-L1 statuses, significant differences in the number of mutations were not found, but there were differences in the distribution of single mutations among patients depending on the IHC status of the tumor. In the case of HER2-positive tumor status, an increase in the number of patients with *TP53* mutations was shown. At the same time, in the case of PD-L1-positive tumor status, an increase in the percentage of tumors with *KRAS, TP53, PIK3CA, PTEN,* and *ERBB* mutations was detected.

### 3.3. Expression of Molecular Markers in Cancers and Association with the Genetic Markers

We identified biological features of gastric cancer associated with the presence of mutations that have a connection with response to therapy (Table 4). In the presence of *PIK3CA* mutation, a 5.6-fold increase in PD-1 expression was detected. In addition, in the presence of *TP53* mutation, a 2.8-fold increase in VEGFR2 expression was shown, and in the presence of *PTEN* mutation, a 3.5-fold increase in mTOR expression and a 3.3-fold decrease in VEGFR2 mRNA level were shown. In the presence of PIK3CA mutation, a 278.5-fold decrease in NF-kB p65 expression was detected.

## 4. Discussion

The altered genes in GCs have the potential to alter biological processes and the proliferative potential of cancer. TP53 gene status predicts clinicopathological features and survival in GC patients [22]. Mutations in PI3K/AKT pathway genes and amplifications of PIK3CA are associated with patterns of recurrence in GCs [23]. PTEN loss is essential in gastric carcinogenesis, and its impact on the PI3K/AKT pathway results in autophagy, cell cycle activation, and metastasis [24]. 

In one study, the most aggressive behavior was found for cancers with *KRAS* mutations [25]. Elsewhere, it has been shown that SMAD has a prognostic value in GC patients; meanwhile, CTNNB1 mutations have decreased expression of βcatenin, which is associated with poor tumor differentiation and shorter overall survival [26]. But an association was not found between the HER2 status and *ERBB* mutation in GCs [27]. Moreover, patients with MSI-H GC harbored more *KRAS* mutation, PD-L1 positivity, CD8 overexpression, and higher TMB, but less HER2 positivity and TP53 mutation [28]. 

The relationship between the tumor genome and biological behavior is not yet fully understood. However, it has been demonstrated in gastric cancer with bone metastases [12]. Activation of altered signaling cascades, particularly PIK3/AKT/mTOR, was combined with the identified changes, making them potential targets for antitumor therapy [13,14]. In this study, it was observed that there is an increase in PD-1 receptor expression when there is *PIK3CA* mutation present, which could be accompanied by changes in tumor immunogenic properties.

The change of molecular factors associated with neoangiogenesis has been shown in cases of *TP53* mutation and *PTEN* mutation, and it is known that the most severe clinical features in cancers are the effects of essential genetic defects. We found that *TP53*-mutation-dependent activation of the proliferation rate and angiogenesis are powerful steps in oncogenesis [22]. Meanwhile, PTEN loss is the predictable result of gene alteration leading to AKT/mTOR overexpression.

In a previous study, the proliferation and metastasis of GC cells were significantly enhanced by ectopic expression of RPS15A, both in vitro and in vivo. RPS15A overexpression also promoted the epithelial–mesenchymal transition (EMT) phenotype formation of GC cells. RPS15A was found to activate the NF-kB signaling pathway by triggering the nuclear translocation and phosphorylation of the p65 NF-kB subunit, transactivating the NF-kB reporter, and elevating target genes of this pathway, based on studies of the underlying mechanisms. RPS15A overexpression led to activation of the Akt/IKK signaling axis in GC cells, while RPS15A knockdown prevented the Akt/IKK signaling axis. The authors’ findings indicate that RPS15A activates the NF- kB pathway through the Akt/IKK signaling axis, resulting in EMT and GC metastasis [29].

Excessive GC growth and poor prognosis of GC are caused by downregulation of the ERK-P65-miR23a/27a/24 axis as a result of low gastrin, as we found in this study. Gastrin acts as a regulator of gastric cancer growth and activates the ERK-P65-miR23a/27a/24 pathway [30]. 

It has been confirmed that the RPS15A/Akt/IKK/NF-kB signaling pathway is effective in combating tumors. In this study, the tumor process was stabilized without further metastasis due to a significant decrease in the NF-kB p65 subunit expression level thanks to PIK3CA inactivating mutations that downregulated the AKT expression level. However, Zu L. D. and his co-authors’ [30] data suggest that downregulation of the NF-kB p65 subunit through the ERK-P65-miR23a/27a/24 signaling pathway can have negative consequences, which should be taken into account.

The availability of significant genetic markers does not ensure the effectiveness of combined treatment in GCs; instead, we found differences in the spectrums of mutations. Furthermore, in patients with no mutations in the targeted gene panel, the best response to treatment and development of complete regression were observed. Meanwhile, the prevalence of TP53 gene mutations was revealed in HER2-positive tumors, and a single mutational variety was found to be associated with the status of PD-L1-positive tumors. These findings lead us to propose that significant genetic markers can affect the biological properties of tumors and influence antitumor benefits by determining cancer’s molecular features.

## 5. Conclusions

The availability of significant genetic markers in GC does not have any impact on the effectiveness of combined treatment. Different mutation spectrums were observed, and patients with an absence of known oncogenic markers showed the best response to treatment and complete regression. Nonetheless, the biological properties of tumors can be altered by significant genetic markers, which can shape the effectiveness of antitumor treatment. Designing personalized therapy depends on IH tumor status, but research has confirmed the association of HER2 with a *TP53* mutation, as well as the immunoheterogeneity of tumors with a PD-L1 status and high mutational diversity. These findings are preliminary and require further confirmation.

## Figures and Tables

**Table 1:** Clinical characteristics of patients.

Indicator	*n* (%)
cT	
cT2	2 (9.5%)
cT3	12 (57.2%)
cT4	7 (33.3%)
cN	
cN0	11 (51.7)
cN1	6 (28.6%)
cN2	4 (19.7%)
pT	
pT0	6 (28.6%)
pT1	5 (23.8%)
pT2	3 (14.2%)
pT4	7 (33.3%)
pN	
pN0	17 (81.0%)
pN1	2 (9.5%)
pN2	2 (9.5%)
Response to treatment	
Regression	14 (66.6%)
Stabilization	7 (33.4)
Targeted sequencing	
No significant genetic markers associated with response to therapy or tumor phenotype	6 (33.3%)
	2 full regressions (33.3%)
	4 partial regressions (66.7%)
Availability of markers	15 (66.7%)

**Table 2:** FLOT regimen’s effectiveness and genetic markers.

Tumor Regression	*n* (%)	Stabilization	*n* (%)
	7 (50.0%)—0 mutations		8 (53.0%)—0 mutations
	7 (50.0%)—9 mutations		7 (47.0%)—11 mutations
χ^2^, *p* > 0.05			
Distribution of mutations associated with treatment response	*KRAS* mutation (chr12:g.25398284C>T)—0.0%	Distribution of mutations associated with treatment response	*KRAS* mutation (chr12:g.25398284C>T)—9.0%
	*PIK3CA* mutation (chr3:g.178952085A>G chr3:g.178936091G>A)—22.2%		*PIK3CA* mutation (chr3:g.178952085A>G chr3:g.178936091G>A)—18.2%
Distribution of mutations associated with phenotype	*TP53* mutation—44.4%	Distribution of mutations associated with phenotype	*TP53* mutation—27.3%
	*PIK3C* mutation—0.0%		*PI3K* mutation—9.0%
	*PTEN* mutation—11.1%		*PTEN* mutation—9.0%
	*ERBB* mutation—11.1%		*ERBB* mutation—0.0%
	*MAPK* mutation—11.1%		*MAPK* mutation—0.0%
	*SMAD4* mutation—0.0%		*SMAD4* mutation—0.0%
χ^2^, *p* < 0.05			

**Table 3:** HER2 and PD-L1 status of the tumor and genetic markers.

HER2 Status	*n (%)*		PD-L1 Status	*n (%)*	
	17 (81.0%)—negative status			16 (76.0%)—negative status	
	4 (19.0%)—positive status			5 (24.0%)—positive status	
	χ^2^, *p* > 0.05				
Distribution of mutations associated with treatment response	positive status	negative status	Distribution of mutations associated with treatment response	positive status	negative status
	*KRAS* (chr12:g.25398284C>T)—0 (0%)	*KRAS* (chr12:g.25398284C>T)—1 (7.2%)		*KRAS*—1 (20.0%) (chr12:g.25398284C>T)	*KRAS* (chr12:g.25398284C>T)—0 (0.0%)
	*PI3KCA* mutation (chr3:g.178952085A>G chr3:g.178936091G>A)—0 (0.0%)	mutation *PIK3C* (chr3:g.178952085A>G chr3:g.178936091G>A)—4 (28.5%)		*PI3KCA* (chr3:g.178952085A>G chr3:g.178936091G>A)—0 (0%)	*PI3KCA* (chr3:g.178952085A>G chr3:g.178936091G>A)—3 (23.0%)
Distribution of mutations associated with phenotype	*TP5*3—2 (50.0%)	*TP5*3—5 (35.5%)	Distribution of mutations associated with phenotype	*TP53*—1 (20%)	*TP53*—7 (53.9%)
	*PIK3CA*—0 (0.0%)	*PIK3CA*—1 (7.2%)		*PIK3CA*—1 (20%)	*PIK3CA*—1 (7.7%)
	*PTEN*—0 (0.0%)	*PTEN*—2 (14.4%)		*PTEN*—1 (20%)	*PTEN*—1 (7.7%)
	*ERBB*—0 (0.0%)	*ERBB*—1 (7.2%)		*ERBB*—1 (20%)	*ERBB*—1 (7.7%)
	χ^2^, *p* < 0.05			χ^2^, *p* < 0.05	

**Table 4:** Molecular markers and GC mutations associated with response to therapy and tumor phenotype.

	Mutations Associated with Treatment Response		Mutations Associated with Phenotype					
	Mutation PIK3CA		Mutation TP53		Mutation PIK3CA		Mutation PTEN	
	Mutation-Free, *n* = 17	Have Mutations, *n* = 4	Mutation-Free, *n* = 13	Has a Mutation, *n* = 8	Mutation-Free, *n* = 18	Has a Mutation, *n* = 3	Mutation-Free, *n* = 18	Has a Mutation, *n* = 3
4EBP1, Relative Unit	0.5 (0.24; 9.25)	14.50 (0.27; 18.18)	0.50 (0.24; 9.25)	0.86 (0.25; 4.46)	0.80 (0.27; 4.56)	0.32 (0.18; 4.00)	0.80 (0.27; 4.35)	0.32 (0.18; 14.50)
AKT, Relative Unit	0.63 (0.48; 65.08)	3.10 (0.60; 20.00)	0.63 (0.48; 65.08)	1.83 (0.46; 3.51)	0.99 (0.50; 3.10)	0.66 (0.18; 128.00)	0.66 (0.50; 2.64)	128.00 (0.18; 200.00)
c-RAF Relative Unit	0.73 (0.22; 1.30)	1.00 (0.24; 1.88)	0.73 (0.22; 1.30)	1.14 (0.54; 1.78)	1.00 (0.41; 1.42)	0.13 (0.12; 1.44)	0.88 (0.30; 1.27)	1.44 (0.13; 1.88)
GSK-3β Relative Unit	0.69 (0.18; 4.17)	0.97 (0.13; 16.39)	0.69 (0.18; 4.17)	0.86 (0.16; 2.00)	0.79 (0.18; 1.74)	0.23 (0.01; 512.00)	0.75 (0.18; 1.19)	16.39 (0.01; 512.00)
70S 6 kinase, Relative Unit	1.03 (0.48; 1.34)	0.98 (0.47; 2.40)	1.03 (0.48; 1.34)	0.96 (0.33; 2.25)	1.07 (0.54; 1.87)	0.27 (0.13; 1.44)	0.98 (0.47; 1.38)	1.44 (0.13; 2.40)
m-TOR, Relative Unit	1.13 (0.60; 1.92)	1.32 (0.50; 3.95)	1.13 (0.60; 1.92)	1.43 (0.17; 5.12)	1.11 (0.50; 1.84)	2.00 (1.15; 47158.39)	1.11 (0.50; 1.53)	3.95 (2.00; 47.39) ##
PDK1, Relative Unit	0.84 (0.14; 2.42)	2.20 (0.25; 4.55)	0.84 (0.14; 2.42)	1.06 (0.47; 2.10)	1.24 (0.76; 2.20)	0.03 (0.01; 0.71)	0.93 (0.71; 2.01)	0.03 (0.01; 4.55)
PTEN, Relative Unit	0.44 (0.13; 2.82)	3.97 (0.16; 8.00)	0.44 (0.13; 2.82)	0.17 (0.01; 3.21)	0.54 (0.16; 3.73)	0.01 (0.01; 0.33)	0.33 (0.16; 2.69)	0.01 (0.01; 3.97)
NF-kB p65, Relative Unit	0.28 (0.11; 1.06)	1.37 (0.13; 1.53)	0.28 (0.11; 1.06)	0.78 (0.12; 1.90)	0.73 (0.13; 1.44)	0.0028 (0.00; 0.29) #	0.33 (0.13; 1.44)	0.0028 (0.00; 1.37)
NF-kB p50, Relative Unit	1.21 (0.25; 6.85)	5.70 (0.16; 8.00)	1.21 (0.25; 6.85)	0.60 (0.13; 4.56)	1.00 (0.19; 5.70)	0.38 (0.13; 23579.19)	0.68 (0.19; 2.95)	5.70 (0.13; 23.19)
VEGFR2, Уcл. Eд.	0.74 (0.12; 2.33)	2.00 (1.07; 2.21)	0.74 (0.12; 2.33)	2.11 (1.26; 4.27000 **)	1.73 (0.46; 2.65)	0.06 (0.00; 3.48)	2.00 (0.46; 3.48)	0.06 (0.00; 1.07) ##
VEGF, Relative Unit	0.99 (0.37; 1.84)	1.04 (0.91; 4.48)	0.99 (0.37; 1.84)	0.46 (0.02; 6.11)	0.88 (0.03; 1.71)	0.93 (0.01; 2.00)	0.88 (0.03; 1.09)	2.00 (0.01; 4.48)
CAIX, Relative Unit	0.64 (0.20; 6.44)	4.00 (0.19; 8.88)	0.64 (0.20; 6.44)	0.36 (0.23; 2.60)	0.47 (0.20; 4.00)	0.35 (0.19; 11.51)	0.37 (0.20; 2.44)	8.88 (0.19; 11.51)
HIF-1, Relative Unit	0.88 (0.27; 14.01)	7.59 (0.13; 23.63)	0.88 (0.27; 14.01)	0.98 (0.08; 1.45)	0.96 (0.13; 7.59)	0.72 (0.03; 2.30)	0.96 (0.13; 2.30)	0.72 (0.03; 23.63)
HIF-2, Relative Unit	0.43 (0.11; 2.37)	1.13 (0.13; 4.51)	0.43 (0.11; 2.37)	0.66 (0.09; 2.77)	0.61 (0.09; 3.36)	0.36 (0.20; 0.50)	0.20 (0.09; 2.17)	0.50 (0.36; 4.51)
VHL, Relative Unit	0.55 (0.21; 1.26)	0.25 (0.24; 6.00)	0.55 (0.21; 1.26)	0.19 (0.05; 1.12)	0.38 (0.12; 1.36)	0.36 (0.16; 1.15)	0.38 (0.12; 1.15)	0.36 (0.16; 6.00)
PD-1, Relative Unit	0.75 (0.29; 1.95)	2.58 (0.92; 32.00 *)	0.75 (0.29; 1.95)	0.38 (0.06; 1.04)	0.68 (0.07; 1.15)	0.50 (0.18; 1.32)	0.68 (0.07; 1.15)	0.50 (0.18; 2.58)
PD-L1, Relative Unit	0.44 (0.33; 0.93)	3.87 (0.50; 7.79)	0.44 (0.33; 0.93)	2.21 (0.58; 5.66)	0.84 (0.35; 3.01)	0.53 (0.06; 0.81)	0.81 (0.35; 1.41)	0.53 (0.06; 7.79)
PD-L2, Relative Unit	0.37 (0.22; 1.05)	3.47 (0.25; 5.47)	0.37 (0.22; 1.05)	0.78 (0.38; 11.96)	0.60 (0.33; 2.46)	0.18 (0.13; 2.14)	0.60 (0.33; 1.79)	0.18 (0.13; 5.47)
AMPK, Relative Unit	0.68 (0.19; 1.67)	1.27 (0.47; 4.00)	0.68 (0.19; 1.67)	1.29 (0.30; 1.66)	1.21 (0.47; 1.87)	0.29 (0.09; 1.44)	1.21 (0.29; 1.87)	0.47 (0.09; 1.44)
LC3B, Relative Unit	0.57 (0.16; 4.95)	1.89 (0.12; 8.00)	0.57 (0.16; 4.95)	0.38 (0.10; 3.23)	0.60 (0.12; 3.97)	0.18 (0.13; 0.30)	0.50 (0.12; 3.97)	0.18 (0.13; 1.89)

*—Significance of differences compared to tumors without PI3K mutation (mutation-free) (chr3:g.178952085A>G chr3:g.178936091G>A); **—significance of differences compared to tumors without TP53 mutation; #—significance of differences compared to tumors without PIK3CA mutation; ##—significance of differences compared to tumors without PTEN mutation.

## Data Availability

The data presented in this study are available on request from the corresponding author. The data are not publicly available due to privacy or ethical restrictions.
